# Generalizability and reach of a randomized controlled trial to improve oral health among home care recipients: comparing participants and nonparticipants at baseline and during follow-up

**DOI:** 10.1186/s13063-022-06470-y

**Published:** 2022-07-08

**Authors:** Jonas Czwikla, Alexandra Herzberg, Sonja Kapp, Stephan Kloep, Heinz Rothgang, Ina Nitschke, Cornelius Haffner, Falk Hoffmann

**Affiliations:** 1 Department of Health Services Research, Carl von Ossietzky University of Oldenburg, Ammerländer Heerstraße 114-118, 26129 Oldenburg, Germany; 2 Department of Health, Long-Term Care and Pensions, SOCIUM Research Center on Inequality and Social Policy, University of Bremen, Mary-Somerville-Straße 5, 28359 Bremen, Germany; 3 High-Profile Area of Health Sciences, University of Bremen, Bibliothekstraße 1, 28359 Bremen, Germany; 4 Competence Center for Clinical Trials, University of Bremen, Linzer Straße 4, 28359 Bremen, Germany; 5 Division of Gerodontology, Clinic of Prosthetic Dentistry and Dental Materials Science, University Medical Center, Liebigstraße 10-14, 04103 Leipzig, Germany; 6 Clinic of General, Special Care and Geriatric Dentistry, Center of Dental Medicine, University of Zurich, Plattenstraße 11, CH-8032 Zurich, Switzerland; 7 Special Care- and Geriatric Dentistry, Städtisches Klinikum Harlaching München, Sanatoriumsplatz 2, 81545 Munich, Germany

**Keywords:** Generalizability, Randomized controlled trial, Reach, Non-response, Oral health, Geriatric dentistry, Long-term care, Home care, Claims data

## Abstract

**Background:**

The generalizability of randomized controlled trials (RCTs) with a low response can be limited by systematic differences between participants and nonparticipants. This participation bias, however, is rarely investigated because data on nonparticipants is usually not available. The purpose of this article is to compare all participants and nonparticipants of a RCT to improve oral health among home care recipients at baseline and during follow-up using claims data.

**Methods:**

Seven German statutory health and long-term care insurance funds invited 9656 home care recipients to participate in the RCT *MundPflege*. Claims data for all participants (*n* = 527, 5.5% response) and nonparticipants (*n* = 9129) were analyzed. Associations between trial participation and sex, age, care dependency, number of Elixhauser diseases, and dementia, as well as nursing, medical, and dental care utilization at baseline, were investigated using multivariable logistic regression. Associations between trial participation and the probability of (a) moving into a nursing home, (b) being hospitalized, and (c) death during 1 year of follow-up were examined via Cox proportional hazards regressions, controlling for baseline variables.

**Results:**

At baseline, trial participation was positively associated with male sex (odds ratio 1.29 [95% confidence interval 1.08–1.54]), high (vs. low 1.46 [1.15–1.86]) care dependency, receiving occasional in-kind benefits to relieve caring relatives (1.45 [1.15–1.84]), having a referral by a general practitioner to a medical specialist (1.62 [1.21–2.18]), and dental care utilization (2.02 [1.67–2.45]). It was negatively associated with being 75–84 (vs. < 60 0.67 [0.50–0.90]) and 85 + (0.50 [0.37–0.69]) years old. For morbidity, hospitalizations, and formal, respite, short-term, and day or night care, no associations were found. During follow-up, participants were less likely to move into a nursing home than nonparticipants (hazard ratio 0.50 [0.32–0.79]). For hospitalizations and mortality, no associations were found.

**Conclusions:**

For half of the comparisons, differences between participants and nonparticipants were observed. The RCT’s generalizability is limited, but to a smaller extent than one would expect because of the low response. Routine data provide a valuable source for investigating potential differences between trial participants and nonparticipants, which might be used by future RCTs to evaluate the generalizability of their findings.

**Trial registration:**

German Clinical Trials Register DRKS00013517. Retrospectively registered on June 11, 2018.

**Supplementary Information:**

The online version contains supplementary material available at 10.1186/s13063-022-06470-y.

## Background

Randomized controlled trials (RCTs) can ensure high internal validity (i.e., the observed effect of an intervention is likely to be correct within the study population) and are therefore the gold standard for the evaluation of an intervention’s effectiveness [1]. However, their generalizability (also termed as external validity, i.e., the degree to which the observed effect of an intervention is correct within the entire target population) is often queried. Especially a low response makes RCTs prone to systematic differences between participants and nonparticipants [2, 3]. This is particularly relevant for complex interventions and in populations with difficult recruitment conditions, such as home care recipients, a population group typically suffering from multimorbidity, frailty, cognitive impairments, and polypharmacy, which has been increasing in most countries due to demographic aging [4–6]. If systematic differences between participants and nonparticipants exist, the intervention under study may have a different degree of effectiveness when offered as usual care. This participation bias (also known as non-response bias), however, is rarely investigated because data on the group of nonparticipants is usually not available in RCTs [7, 8].

Routinely collected data is increasingly used for the recruitment and outcome assessment of RCTs [9–12], which resulted in the development of the Consolidated Standards of Reporting Trials (CONSORT) extension for RCTs conducted using cohorts and routinely collected data (CONSORT-ROUTINE) [13]. For examining participation bias, administrative databases, electronic health records, and registries provide a valuable data source because they typically comprise information for all participants and nonparticipants [14–20]. For example, when recruiting individuals from a population being insured with a health insurance fund, the data comprises detailed information on demographic characteristics, diagnoses, and medical care utilization for all participants and nonparticipants, both at baseline and during follow-up. This is a major advantage over RCTs using short questionnaires or personal interviews to assess at least some cross-sectional information for a selective group of nonparticipants agreeing to be surveyed [21–27]. A further benefit of non-response analyses based on routinely collected data is that they offer the opportunity to identify the potential for improving the recruitment of RCTs and reach of interventions [19, 28].

The purpose of this article is to investigate whether participation in a RCT of a complex intervention to improve oral health among home care recipients is associated with sex, age, care dependency, and morbidity, as well as nursing, medical, and dental care utilization at baseline using claims data. Associations between trial participation and the probability of (a) moving into a nursing home, (b) being hospitalized, and (c) death during follow-up are also examined.

## Methods

### Randomized controlled trial

The two-arm RCT *MundPflege* was approved by the University of Bremen Ethics Committee on 21 March 2018 (reference number “MundPflege”). The detailed methodology and the main findings of the trial have been published elsewhere [6]. In brief, seven German statutory health and long-term care (LTC) insurance funds, which have the same mandate to finance medical and nursing care and follow the same rules, invited all eligible home care recipients to participate. Individuals were eligible if they were (i) insured with one of the cooperating insurance funds, (ii) at least 18 years old, (iii) a home care recipient according to the German Social Code Book XI (i.e., a person whose LTC dependency was legally evaluated by the Medical Advisory Service of the statutory health insurance and who receives LTC benefits in the home care setting), and (iv) a resident in the Free Hanseatic City of Bremen or the federal state of Lower Saxony. Between the first and second quarters of 2018, the insurance funds identified all insured persons who met these inclusion criteria. The invitation letter and one reminder were sent out by the insurance funds in the second quarter of 2018 (hereinafter referred to as the baseline quarter). The letters informed about the importance of oral health among LTC dependents and the design of the RCT. The invited persons were asked to provide informed consent for trial participation. They were briefed that they will be randomized either into the treatment (50%) or control (50%) group, whereby only the former will receive an oral health intervention by dentists in addition to usual care at *t*_0_. The intervention comprised an oral health assessment, dental treatment recommendations, and oral health education in the patient’s domicile. The invited persons were also informed that both the treatment and control group participants will receive a blinded outcome assessment at *t*_1_ [6].

**Fig. 1:**
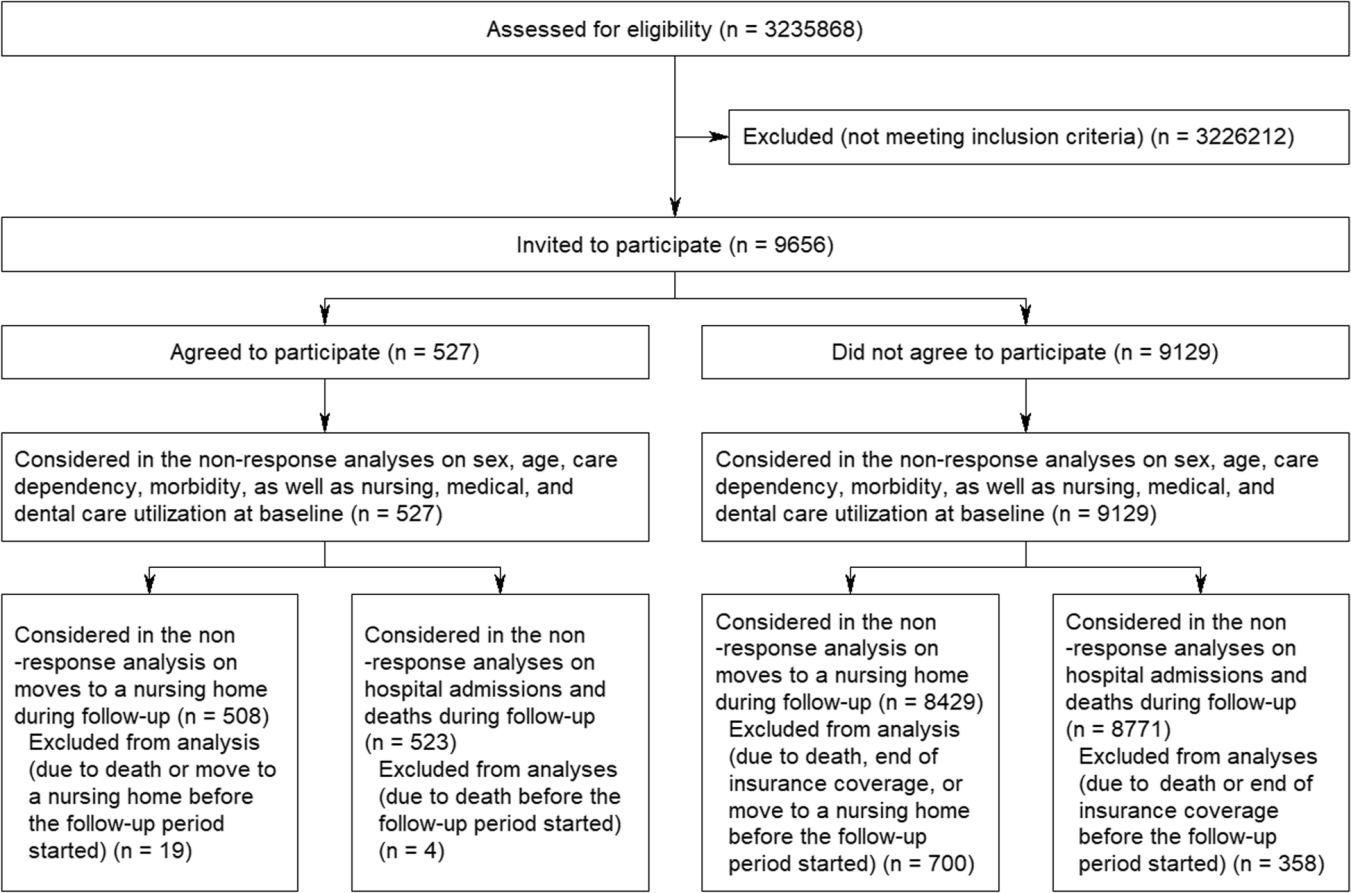
Flow diagram

Of 3.2 million insured persons, 9656 met the inclusion criteria and were invited to participate (Fig. 1) [6]. Among the invited home care recipients, 527 (5.5% response, 259 treatment group and 268 control group participants) participated and 9129 were nonparticipants. In the treatment group, 164 participants received the intervention between May 2018 and November 2019 (*t*_0_). The oral health status of 112 treatment group (56.8% dropouts) and 137 control group (48.9% dropouts) participants was assessed between January 2019 and November 2020 (*t*_1_). The comparison of the baseline characteristics between the treatment group and control group participants whose outcomes were assessed is described elsewhere and indicated no differences [6]. After a mean follow-up time of more than 1 year, although not statistically significant, the intervention tended to improve objective oral health, which was the primary outcome. Regarding the secondary outcomes, the intervention significantly reduced the prevalence of any periodontal problems, while no effectiveness was observed regarding subjective oral health and the prevalence of periodontitis [6].

To guide reporting of this article, we followed CONSORT-ROUTINE [13] (Additional file 1).

### Claims data

For the non-response analysis, health and LTC insurance claims data were available for all participants and nonparticipants of the RCT. The information which home care recipients were invited to participate in the RCT was provided by the insurance funds, and the information which individuals participated in the trial was obtained from the Competence Center for Clinical Trials of the University of Bremen, the trial’s trusted third party. These data were linked to the claims data via a unique person identifier.

The analyzed claims data covered the period from the third quarter of 2017 to the second quarter of 2019. The *baseline period* of the non-response analysis comprised the three quarters before the baseline quarter and the baseline quarter. The *follow-up period* of the non-response analysis included the four quarters following the baseline quarter.

Besides insurance periods, the claims data comprised information on sex, year of birth, LTC grades, outpatient diagnoses, nursing care utilization, and medical and dental care utilization. The data on the year of birth was used to define the age in the baseline quarter. The data on LTC grades in the baseline quarter differentiates into 5 LTC grades (grade 1 = low limitations, grade 2 = substantial limitations, grade 3 = severe limitations, grade 4 = very severe limitations without special challenges for nursing care, grade 5 = very severe limitations with special challenges for nursing care) [29]. All diagnoses were coded according to the International Classification of Diseases, 10th Revision (ICD-10). The outpatient diagnoses were used to obtain information on the 31 diseases of the Elixhauser comorbidity measure and dementia in the baseline period. The Elixhauser comorbidity measure comprises a broad range of diseases that can be identified based on administrative data and is commonly used in health services research. The 31 diseases were defined using the ICD-10 codes provided by Quan et al. [30]. Dementia, which is not included in the Elixhauser list, was defined based on the ICD-10 codes used by Hoffmann et al. [31]. The information on nursing care utilization comprised information on occasional in-kind benefits to relieve caring relatives, formal care in addition to informal care, respite care by a substitute when relatives are on holiday or sick, short-term care in an institution, and day or night care in an institution during the baseline period [29]. It also comprised information on moves to a nursing home during the baseline and follow-up period. The data on medical care utilization included information on referrals by general practitioners (GPs) to medical specialists during the baseline period which served as a proxy for the coordination of medical care by GPs. It also comprised information on hospital admissions during the baseline and follow-up period. The data on dental care utilization during the baseline period was coded according to the German uniform assessment standard for dental care (BEMA) parts 1 to 5 (part 1 = conservative and surgical treatment and X-ray examinations, part 2 = treatment of injuries of the viscerocranium [jaw fracture], jaw joint disorders [occlusal splints], part 3 = orthodontic treatment, part 4 = systemic treatment of periodontal diseases, part 5 = provision of dentures and crowns) [32, 33]. Information on mortality during the baseline and follow-up period was obtained from insurance periods ended due to death. Information on terminated insurance coverages during the baseline and follow-up period was also obtained from insurance periods.

### Statistical analysis

In the first step, all baseline characteristics available in the claims data were compared between participants and nonparticipants using the chi-square test (expected frequency in all cells of a contingency table ≥ 5), Fisher’s exact test (expected frequency in at least one cell of a contingency table < 5), and nonparametric Wilcoxon-Mann–Whitney test. For all comparisons, the absolute difference between participants and nonparticipants and the corresponding 95% confidence interval (CI) was also calculated. The distributions of sex (female, male), age groups (< 60 [adults], 60–74 [youngest-old], 75–84 [middle-old], 85 + years [oldest-old]), and LTC grades (1/2 [less severe limitations], 3 [severe limitations], 4/5 [very severe limitations]) as well as the mean age were compared. With respect to morbidity, the prevalence of the 31 Elixhauser diseases (0–2 [quartile 1], 3–4 [quartile 2], 5–6 [quartile 3], 7 + [quartile 4] diseases) and dementia were compared. With regard to nursing care, the proportions of home care recipients who utilized the different types of nursing care were compared between participants and nonparticipants. Regarding medical care, the proportions of individuals having a referral by a GP to a medical specialist and being hospitalized were compared. With respect to dental care, the proportion of individuals who utilized the different dental treatments was compared.

Second, a multivariable logistic regression was applied. In this regression, participation in the RCT served as the dependent variable. The independent variables were sex, age group, LTC grade, the unweighted number of Elixhauser diseases, dementia, occasional in-kind benefits to relieve caring relatives, formal care, referral by a GP to a medical specialist, hospital admission, and dental treatment. Respite care by a substitute, short-term care in an institution, and day or night care in an institution were not considered as independent variables to avoid multicollinearity.

In the third step, the proportions of home care recipients who (a) moved into a nursing home, (b) were admitted to a hospital, and (c) died during follow-up were compared between participants and nonparticipants using Kaplan–Meier plots. The outcomes move to a nursing home and death were investigated because moves to a nursing home and deaths were reasons for the dropout of participants in the RCT *MundPflege*. The outcome hospital admission was examined because hospital admissions made it difficult to schedule appointments for the provision of the intervention and for the outcome assessment. The time scale in the Kaplan–Meier plots was time in days since 1 July 2018 (i.e., the first day after the baseline quarter) until the day of the respective outcome, of the end of insurance coverage, or of the end of the follow-up (i.e., 30 June 2019).

Finally, three Cox proportional hazards regressions were applied. In these regressions, move to a nursing home, hospital admission, and death were considered as the dependent variable. In each model, the explanatory variable of interest was trial participation. The time scale was the same as in the Kaplan–Meier plots (i.e., time in days since 1 July 2018 until the day of the respective outcome, of the end of insurance coverage, or of the end of the follow-up on 30 June 2019). Cox regressions were performed including all independent variables considered in the logistic regression as covariates.

The statistical analysis was conducted using SAS 9.4 (SAS Institute Inc., Cary, NC, USA).

## Results

### Descriptive findings on sex, age, care dependency, and morbidity, as well as nursing, medical, and dental care utilization at baseline

The descriptive comparisons at baseline considered all 527 participants and 9129 nonparticipants (Fig. 1). The proportion of men was higher and the mean age lower among participants than among nonparticipants (Table 1). The percentage distribution of LTC grades also differed showing a greater proportion of higher LTC grades in the group of participants compared to the group of nonparticipants. For the number of Elixhauser diseases, no significant differences were found. Regarding the 31 single Elixhauser diseases, only the prevalence of paralysis and the prevalence of other neurological disorders differed (Additional file 1). With respect to dementia, the prevalence was lower among participants compared to nonparticipants.

**Table 1:** Baseline characteristics of the invited home care recipients

Category	Participants (*n* = 527), %	Nonparticipants (*n* = 9129), %	*p*-value	Difference (95% CI)
**Sex**				
Male	49.9	41.3	**.0001**	**8.6 (4.2 to 13.0)**
**Age group**				
< 60 years	19.5	12.9		**6.6 (3.2 to 10.1)**
60–74 years	20.3	16.3		**4.0 (0.4 to 7.5)**
75–84 years	36.6	35.7		0.9 (-3.3 to 5.1)
85 + years	23.5	35.0	** < .0001**	** − 11.5 (− 15.2 to − 7.7)**
Mean (SD)	72.4 (17.4)	76.5 (16.0)	** < .0001**	** − 4.2 (− 5.6 to − 2.7)**
**Long-term care grade**				
1/2	49.9	53.3		− 3.4 (− 7.8 to 1.0)
3	28.8	30.1		− 1.3 (− 5.2 to 2.7)
4/5	21.3	16.6	**.0198**	**4.7 (1.1 to 8.3)**
**Number of Elixhauser diseases**				
0–2	20.3	21.3		− 1.0 (− 4.5 to 2.5)
3–4	28.8	27.6		1.2 (− 2.7 to 5.2)
5–6	21.4	23.8		− 2.3 (− 6.0 to 1.3)
7 +	29.4	27.3	.4818	2.1 (− 1.9 to 6.1)
Mean (SD)	5.2 (3.0)	5.0 (2.9)	.2502	0.2 (− 0.1 to 0.5)
**Dementia**				
Yes	39.7	46.4	**.0024**	** − 6.8 (− 11.1 to − 2.5)**
**Nursing care utilization**				
Occasional in-kind benefits to relieve caring relatives	81.0	76.3	**.0129**	**4.7 (1.3 to 8.2)**
Formal care	28.3	32.0	.0751	− 3.7 (− 7.7 to 0.2)
Respite care by a substitute	30.6	28.7	.3531	1.9 (− 2.2 to 5.9)
Short-term care in an institution	9.9	11.3	.3060	− 1.4 (− 4.1 to 1.2)
Day or night care in an institution	6.3	7.9	.1683	− 1.7 (− 3.8 to 0.5)
**Medical care utilization**				
Referral by a general practitioner to a medical specialist	89.0	83.7	**.0014**	**5.3 (2.5 to 8.0)**
Hospital admission	46.3	43.0	.1325	3.3 (− 1.0 to 7.7)
**Dental care utilization**				
BEMA 1: conservative and surgical treatment and X-ray examinations	69.8	51.5	** < .0001**	**18.4 (14.3 to 22.4)**
BEMA 2: treatment of injuries of the viscerocranium (jaw fracture), jaw joint disorders (occlusal splints)	1.1	1.1	.9453	0.0 (− 0.9 to 1.0)
BEMA 3: orthodontic treatment	0.9	0.1	**.0022**^a^	0.8 (0.0 to 1.6)
BEMA 4: systemic treatment of periodontal diseases	1.1	0.7	.2980^a^	0.4 (− 0.5 to 1.3)
BEMA 5: provision of dentures and crowns	24.3	20.4	**.0317**	**3.9 (0.1 to 7.6)**
BEMA 1–5	70.0	51.6	** < .0001**	**18.4 (14.4 to 22.4)**

*Abbreviations*: *CI* Confidence interval, *SD* Standard deviation, *BEMA* German uniform assessment standard for dental care

Boldface indicates significant differences (*p* < .05; confidence interval not including 0)

^a^*p*-value calculated by using Fisher’s exact test

Regarding nursing care utilization, the proportion of home care recipients who received occasional in-kind benefits to relieve caring relatives at baseline was higher in the group of participants compared to the group of nonparticipants. No significant differences between both groups were observed with regard to the proportions of individuals who utilized formal care, respite care by a substitute, short-term care in an institution, and day or night care in an institution. With respect to medical care utilization, the proportion of individuals with a referral by a GP to a medical specialist was higher among participants than among nonparticipants, whereas no significant difference was observed for hospital admissions. Dental treatments were utilized more frequently by participants than nonparticipants. Regarding the five single dental treatment categories, significant differences were found for the three categories.

### Logistic regression of trial participation at baseline

The adjusted analysis confirmed associations between trial participation and sex, age group, LTC grade, occasional in-kind benefits to relieve caring relatives, referrals by GPs to medical specialists, and dental care utilization, whereas the association between trial participation and dementia was no longer statistically significant (Table 2). Men were more likely to participate than women (odds ratio [OR] 1.29 [95% CI 1.08–1.54]). Moreover, homecare recipients at the age of 75–84 years and 85 + years were less likely to participate compared to those at the age of < 60 years (OR 0.67 [95% CI 0.50–0.90] and 0.50 [95% CI 0.37–0.69], respectively). With respect to LTC grades, individuals with a LTC grade of 4/5 had a higher chance to participate than those with a LTC grade of 1/2 (OR 1.46 [95% CI 1.15–1.86]). Occasional in-kind benefits to relieve caring relatives (OR 1.45 [95% CI 1.15–1.84]), referrals by GPs to medical specialists (OR 1.62 [95% CI 1.21–2.18]), and dental care utilization (OR 2.02 [95% CI 1.67–2.45]) were all positively associated with trial participation.

**Table 2:** Logistic regression analysis of trial participation at baseline (*n* = 9656)

Variable	OR	95% CI
**Sex**		
Male (ref. female)	**1.29**	**(1.08–1.54)**
**Age group (ref. < 60 years)**		
60–74 years	0.78	(0.58–1.05)
75–84 years	**0.67**	**(0.50–0.90)**
85 + years	**0.50**	**(0.37–0.69)**
**Long-term care grade (ref. 1/2)**		
3	1.04	(0.84–1.28)
4/5	**1.46**	**(1.15–1.86)**
**Number of Elixhauser diseases (ref. 0–2)**		
3–4	1.13	(0.87–1.48)
5–6	0.99	(0.74–1.33)
7 +	1.10	(0.83–1.47)
**Dementia**		
Yes (ref. no)	0.90	(0.74–1.11)
**Nursing care utilization**		
Occasional in-kind benefits to relieve caring relatives (ref. no)	**1.45**	**(1.15–1.84)**
Formal care (ref. no)	0.92	(0.74–1.13)
**Medical care utilization**		
Referral by a general practitioner to a medical specialist (ref. no)	**1.62**	**(1.21–2.18)**
Hospital admission (ref. no)	1.09	(0.90–1.31)
**Dental care utilization**		
BEMA 1–5 (ref. no)	**2.02**	**(1.67–2.45)**

*Abbreviations*: *OR* Odds ratio, *CI* Confidence interval, *ref.* reference, *BEMA* German uniform assessment standard for dental care

Boldface indicates significant differences

### Descriptive findings on moves to a nursing home, hospital admissions, and deaths during follow-up

**Fig. 2:**
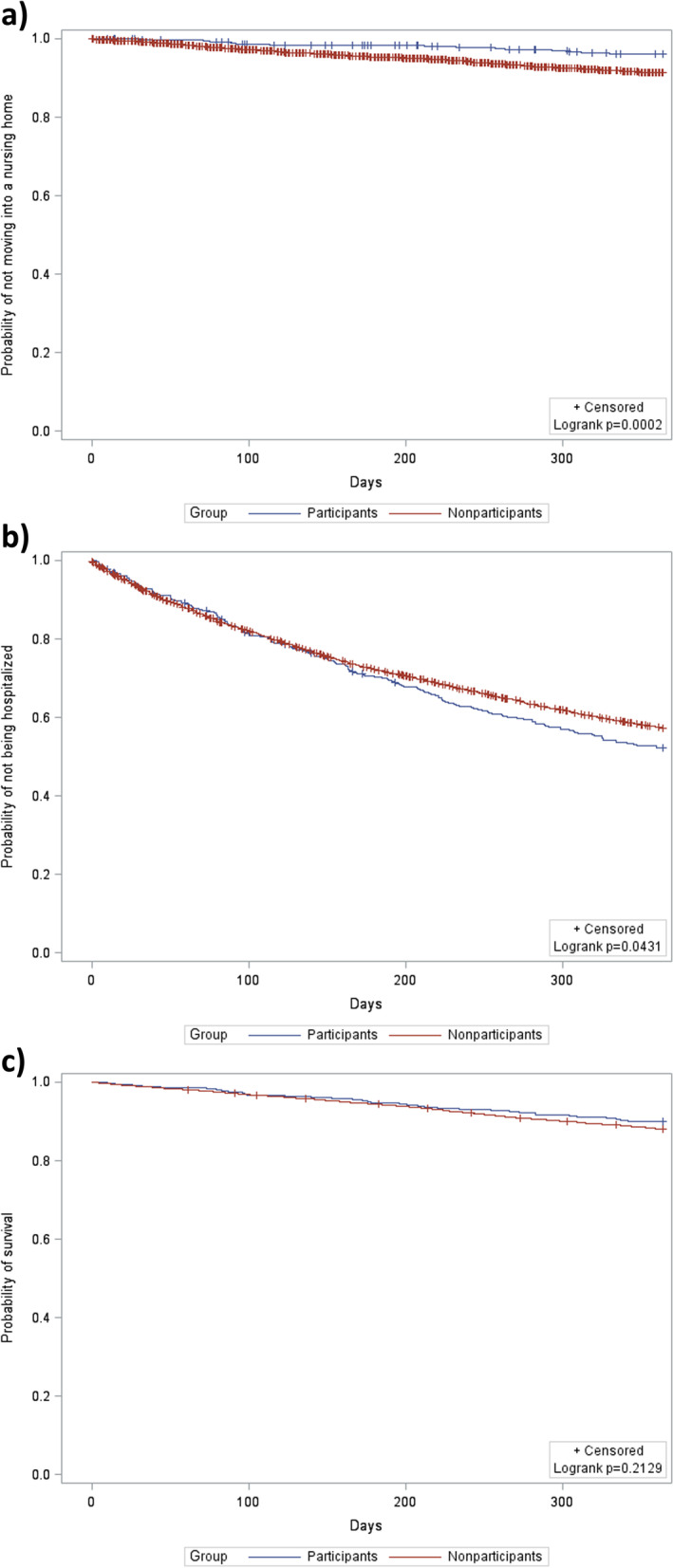
**A** Probability of not moving into a nursing home during follow-up among participants (*n* = 508) and nonparticipants (*n* = 8429). **B** Probability of not being hospitalized during follow-up among participants (*n* = 523) and nonparticipants (*n* = 8771). **C** Probability of survival during follow-up among participants (*n* = 523) and nonparticipants (*n* = 8771)

The analysis on moves to a nursing home during follow-up comprised 508 participants and 8429 nonparticipants (Fig. 1). In this analysis, 19 participants and 700 nonparticipants were not considered because they died (4 participants and 352 nonparticipants), terminated their insurance coverage (6 nonparticipants), or moved into a nursing home (15 participants and 342 nonparticipants) before the follow-up period started. During a mean follow-up time of 330.6 days (SD 84.3 days), 3.7% of the participants and 8.3% of the nonparticipants moved into a nursing home (log rank *p* = 0.0002) (Fig. 2A).

The analyses on hospital admissions and deaths during follow-up comprised 523 participants and 8771 nonparticipants (Fig. 1). In these analyses, only the 362 individuals who died or whose insurance coverage ended before the follow-up period started were not considered. The proportion of home care recipients who were admitted to a hospital during a mean follow-up time of 264.7 days (SD 130.2 days) was 46.8% among participants and 41.9% among nonparticipants (log rank *p* = 0.0431) (Fig. 2B). With respect to mortality, 10.1% of the participants and 12.0% of the nonparticipants died during a mean follow-up time of 342.5 days (SD 68.3 days) (log rank *p* = 0.2129) (Fig. 2C).

### Cox regressions of moves to a nursing home, hospital admissions, and deaths during follow-up

The cox regressions of moves to a nursing home confirmed that participants were less likely to move into a nursing home during follow-up compared to nonparticipants (Table 3). The adjusted hazard ratio (HR) for moving into a nursing home for participants vs. nonparticipants was 0.50 (95% CI 0.32–0.79). Regarding hospital admissions, the difference between participants and nonparticipants was no longer significant (HR 1.12 [95% CI 0.98–1.27]). With respect to mortality, again, no significant difference was observed (HR 0.92 [95% CI 0.70–1.21]).

**Table 3:** Cox regression analyses of moves to a nursing home (*n* = 8937), hospital admissions (*n* = 9294), and deaths (*n* = 9294) during follow-up

Variable	Move to a nursing home		Hospital admission		Death	
	**HR**	**95% CI**	**HR**	**95% CI**	**HR**	**95% CI**
**Participation**						
Yes (ref. no)	**0.50**	**(0.32–0.79)**	1.12	(0.98–1.27)	0.92	(0.70–1.21)
**Sex**						
Male (ref. female)	0.91	(0.78–1.06)	**1.08**	**(1.01–1.15)**	**1.51**	**(1.34–1.70)**
**Age group (ref. < 60 years)**						
60–74 years	**2.01**	**(1.19–3.40)**	**1.41**	**(1.23–1.62)**	**4.13**	**(2.68–6.37)**
75–84 years	**3.36**	**(2.07–5.47)**	**1.63**	**(1.43–1.86)**	**5.55**	**(3.65–8.45)**
85 + years	**5.06**	**(3.12–8.20)**	**1.70**	**(1.48–1.94)**	**9.24**	**(6.09–14.03)**
**Long-term care grade (ref. 1/2)**						
3	**1.40**	**(1.19–1.65)**	**1.15**	**(1.07–1.24)**	**1.51**	**(1.31–1.74)**
4/5	**1.33**	**(1.06–1.68)**	**1.25**	**(1.14–1.38)**	**3.15**	**(2.71–3.67)**
**Number of Elixhauser diseases (ref. 0–2)**						
3–4	0.89	(0.71–1.10)	**1.17**	**(1.06–1.30)**	1.11	(0.91–1.36)
5–6	0.95	(0.76–1.19)	**1.38**	**(1.24–1.54)**	**1.25**	**(1.02–1.54)**
7 +	0.86	(0.68–1.08)	**1.70**	**(1.53–1.89)**	**1.60**	**(1.31–1.95)**
**Dementia**						
Yes (ref. no)	**1.64**	**(1.39–1.93)**	0.99	(0.92–1.06)	1.05	(0.92–1.19)
**Nursing care utilization**						
Occasional in-kind benefits to relieve caring relatives (ref. no)	**5.13**	**(3.57–7.37)**	**1.12**	**(1.03–1.22)**	0.91	(0.79–1.07)
Formal care (ref. no)	**1.86**	**(1.59–2.17)**	1.07	(1.00–1.15)	**1.26**	**(1.10–1.44)**
**Medical care utilization**						
Referral by a general practitioner to a medical specialist (ref. no)	**0.74**	**(0.61–0.91)**	1.08	(0.97–1.19)	0.89	(0.74–1.06)
Hospital admission (ref. no)	**1.36**	**(1.17–1.58)**	**1.84**	**(1.73–1.97)**	**1.68**	**(1.48–1.90)**
**Dental care utilization**						
BEMA 1–5 (ref. no)	0.88	(0.75–1.02)	0.98	(0.92–1.04)	**0.73**	**(0.65–0.82)**

*Abbreviations*: *HR* Hazard ratio, *CI* Confidence interval, *ref.* reference, *BEMA* German uniform assessment standard for dental care

Boldface indicates significant differences

## Discussion

This work compared all participants and nonparticipants of a RCT to improve oral health among home care recipients at baseline and during follow-up. At baseline, differences were found for sex, age, LTC dependency, occasional in-kind benefits to relieve caring relatives, referral by a GP to a medical specialist, and dental care utilization. The largest difference was observed for dental care utilization, whereby participants were more likely to have utilized dental treatments than nonparticipants. No differences were observed for the number of Elixhauser diseases, dementia, and hospitalizations, as well as formal, respite, short-term, and day or night care. During 1 year of follow-up, trial participants were less likely to move into a nursing home than nonparticipants, whereas for hospitalizations and mortality no associations were found.

Regarding generalizability, our non-response analysis indicates that the findings of the RCT *MundPflege* are limited, but to a smaller extent than one would expect because of the low response, which might be explained by our comparatively less personal recruitment strategy [6, 34]. We revealed statistically significant differences between trial participants and nonparticipants for almost half of the comparisons. A relevant limitation of generalizability might result from the difference in dental care utilization at baseline. Assuming that individuals without dental care utilization are more likely to have unmet dental care needs than their counterparts, the intervention might have significantly improved the oral health status if the response among this population group had been greater. Therefore, the intervention could be more effective when offered as usual care.

The findings from our non-response analysis suggest that RCTs with a low response are not generally unsuitable for evaluating the effectiveness of complex interventions to improve health in populations with difficult recruitment conditions. Compared to RCTs which are the gold standard for the evaluation of interventions, non-randomized studies might achieve a higher response but, at the same time, tend to be more prone to selection bias potentially leading to incorrect estimates of effectiveness [35, 36]. In cases of RCTs with a low response, provided that statistical power is still sufficient, their methodological advantages can be exploited when data for all trial participants and nonparticipants is available (e.g., from administrative databases, electronic health records, or registries) which allows a systematic investigation of participation bias [14, 18].

With respect to the reach of the RCT, our non-response analysis shows that men, younger individuals, and those with high LTC dependency, who receive occasional in-kind benefits to relieve caring relatives, with a referral by a GP to a medical specialist, and with dental care utilization, were significantly more likely to participate than their counterparts. In line with a previous study [37], individuals who are not moving into a nursing home during follow-up were also more likely to participate. Because most of the differences between participants and nonparticipants were rather small and no differences for morbidity, formal care, respite care by a substitute, short-term care in an institution, day or night care in an institution, and hospitalizations at baseline as well as hospitalizations and mortality during follow-up were found, there is only little potential for improving the reach of the intervention among certain population groups. As a comparatively big difference between participants and nonparticipants was observed regarding dental care utilization at baseline, an opportunity for improving the reach of the RCT would offer the attempt to promote the participation of home care recipients currently not utilizing dental care. A potential reason for the lower dental care utilization among nonparticipants could be that their relatives are not organizing contacts to dentists although, in Germany, at least one annual contact to a dentist is advised. To increase the reach of the RCT among home care recipients currently not utilizing dental care, involving their GPs and formal caregivers (if applicable) in the recruitment strategy could be a promising approach [6, 34, 38, 39].

Ideally, RCTs have both a high response and representative study population. Barriers and facilitators to trial participation have been described [40–44], and a variety of tools and strategies have been proposed to improve the recruitment to RCTs [45–49]. Furthermore, it could be worthwhile to conduct a pilot trial to estimate the response expectable in the main trial and identify the potential for improvement [50]. This would be in line with the Framework for Developing and Evaluating Complex Interventions by the UK Medical Research Council and National Institute of Health Research, which strongly recommends to assess the feasibility of any parts of a study including recruitment prior to conducting a full-scale evaluation [51]. Moreover, if data for participants and nonparticipants of the pilot trial is available, a non-response analysis in addition to qualitative research could help to inform the recruitment strategy of the main trial. This approach may help to avoid the frequently occurring premature discontinuation or extension of RCTs due to not achieving response targets [52, 53].

Overall, routinely collected data provide a valuable opportunity to improve RCTs. Compared to traditional RCTs, those using routine data have been shown to generate additional insights [54]. However, getting access to data from administrative databases, electronic health records, and registries is often not straightforward [55]. If such data is accessible and considered in a RCT, characteristics should be reported not only for trial participants, but also for nonparticipants, as explicitly stated in CONSORT-ROUTINE item 15 [13]. Overall, the entire CONSORT-ROUTINE checklist should be considered to improve the currently limited reporting quality of RCTs using routinely collected data [9–11].

## Strengths and limitations

The main strength of this work is that a large amount of data was available enabling us to systematically compare all participants and nonparticipants of a RCT at baseline and during follow-up. There are, however, some important limitations. First, the cooperating insurance funds identified all individuals eligible for participation between the first and second quarters of 2018. The invitation letters were, however, sent out in the second quarter of 2018. Due to this time gap of a few weeks, some eligible individuals moved into a nursing home, terminated their insurance coverage, or died before they received the invitation. All these individuals were considered as nonparticipants in our baseline analysis although they actually had no chance to participate. However, this should not have significantly affected our findings assuming that most of these individuals would not have participated in the RCT even if they had received the invitation earlier due to their upcoming move to a nursing home, termination of insurance coverage, or death. A second limitation is that claims data was available only for the first year of follow-up and not for the whole study period of the RCT which, due to the COVID-19 pandemic, ended 17 months later. It is, however, unlikely that the comparison of participants and nonparticipants after more than 1 year of follow-up would have led to other results than our comparison during the first year of follow-up. Third, *t*_0_ and *t*_1_ of the RCT were not considered in the present non-response analysis because information on *t*_0_ was available only for the treatment group participants who received the intervention at *t*_0_ (no information for treatment group dropouts, control group participants, and nonparticipants). Furthermore, information on *t*_1_ was available only for the treatment and control group participants who received the outcome assessment at *t*_1_ (no information for treatment group and control group dropouts, and nonparticipants). We therefore compared participants and nonparticipants only at baseline (i.e., when the invitation letters were sent out) and during a fix time period covering the first year of follow-up (i.e., the year after the invitation letters were sent out). Fourth, the claims data analyzed was originally assessed for billing purposes and includes no information on functional limitations, frailty, restricted cognitive abilities, and medical and dental care needs. LTC grades, however, served as a proxy for this missing information. Furthermore, we were unable to compare outpatient procedures such as vaccinations or other preventive services as well as drug prescriptions between participants and nonparticipants because this information was not provided. Fifth, while the validity of information on sex, age, LTC grades, nursing care utilization, medical and dental care utilization, and mortality is considered high due to their relevance for billing purposes, that of morbidity obtained from outpatient diagnoses might be lower. Sixth, in our descriptive analyses, we applied a large number of statistical tests, and therefore, some of our statistically significant findings might be explained by the problem of multiple testing. However, our adjusted analyses confirmed most of the descriptive findings. Finally, the RCT was conducted in two of 16 German federal states and recruited insured persons from seven of currently 97 statutory health and LTC insurance funds. The representativeness of the invited individuals for all statutorily (90% of the population) and privately (10% of the population) insured persons in Germany as well as for persons in other health care systems might therefore be limited. For example, the proportion of statutorily and privately insured persons differs between regions, and differences between German insurance funds regarding age, sex, educational level, and the prevalence of chronic diseases have been described [56, 57]. However, compared to other recruitment strategies such as recruiting via selected practices, recruiting trial participants from insurance funds can generally be expected to result in a higher representativeness because a larger proportion of the entire target population can be invited.

## Conclusions

For half of the comparisons, differences between participants and nonparticipants of a RCT to improve oral health among home care recipients were observed at baseline and during follow-up. The generalizability of the findings of the RCT are therefore limited, but to a smaller extent than one would expect because of the low response. Routinely collected data provide a valuable data source for the investigation of potential differences between trial participants and nonparticipants, which might be used by future RCTs to evaluate the generalizability of their findings.

## Supplementary Information

**Additional file 1:** **Table S1.** Baseline morbidity of the invited home care recipients. **Table S2.** Combined CONSORT 2010 and CONSORT-ROUTINE Checklist.

## Data Availability

The data that support the findings of this study are available from the insurance funds (i.e., BAHN-BKK, Betriebskrankenkasse der Deutschen Bank, Daimler BKK, energie-BKK, Novitas BKK, pronova BKK, SBK Siemens Betriebskrankenkasse) and Competence Center for Clinical Trials of the University of Bremen, but restrictions apply to the availability of these data, which were used under license for the current study, and so are not publicly available. Therefore, data sharing is only feasible upon reasonable request and in collaboration with the authors, insurance funds, and the Competence Center for Clinical Trials of the University of Bremen.

## Abbreviations

BEMA: German uniform assessment standard for dental care
CI: Confidence interval
GP: General practitioner
HR: Hazard ratio
ICD-10: International Classification of Diseases, 10th Revision
LTC: Long-term care
OR: Odds ratio
RCT: Randomized controlled trial
SD: Standard deviation
