# Supplementary material for: Generalizability and reach of a randomized controlled trial to improve oral health among home care recipients: comparing participants and nonparticipants at baseline and during follow-up
Source: Trials. 2022 Jul 8;23:560. doi: 10.1186/s13063-022-06470-y (PMC9264743; doi:10.1186/s13063-022-06470-y)
Supplement: Supplementary file 1 — Additional file 1: Table S1. Baseline morbidity of the invited home care recipients. Table S2. Combined CONSORT 2010 and CONSORT-ROUTINE Checklist. [file 13063_2022_6470_MOESM1_ESM.pdf]

Additional file 1

Generalizability and reach of a randomized controlled trial to improve oral health among home care recipients: comparing participants and nonparticipants at baseline and during follow-up

Jonas Czwikla, Alexandra Herzberg, Sonja Kapp, Stephan Kloep, Heinz Rothgang, Ina Nitschke, Cornelius Haffner, Falk Hoffmann

Correspondence: Dr. Jonas Czwikla
Carl von Ossietzky University of Oldenburg
Department of Health Services Research
Ammerländer Heerstraße 114-118
26129 Oldenburg, Germany

Email jonas.czwikla@uni-oldenburg.de

Table S1 Baseline morbidity of the invited home care recipients

Category	Participants (n=527) %	Nonparticipants (n=9,129) %	p-value	Difference (95% CI)
**Single Elixhauser diseases**				
Congestive heart failure	27.9	29.1	.5550	-1.2 (-5.1 – 2.7)
Cardiac arrhythmias	34.3	32.9	.4911	1.5 (-2.7 – 5.6)
Valvular disease	18.8	18.2	.7375	0.6 (-2.8 – 4.0)
Pulmonary circulation disorders	5.7	4.3	.1446	1.3 (-0.7 – 3.4)
Peripheral vascular disorders	24.5	24.7	.9125	-0.2 (-4.0 – 3.6)
Hypertension, uncomplicated	75.7	76.2	.8128	-0.5 (-4.2 – 3.3)
Hypertension, complicated	13.7	12.7	.5227	1.0 (-2.1 – 4.0)
Paralysis	19.9	13.9	**.0001**	**6.0 (2.6 – 9.5)**
Other neurological disorders	26.9	18.9	**.0001**	**8.0 (4.2 – 11.9)**
Chronic pulmonary disease	31.3	28.9	.2355	2.4 (-1.7 – 6.5)
Diabetes, uncomplicated	30.0	33.7	.0790	-3.7 (-7.7 – 0.3)
Diabetes, complicated	20.3	21.9	.3954	-1.6 (-5.1 – 2.0)
Hypothyroidism	13.7	13.0	.6619	0.7 (-2.4 – 3.7)
Renal failure	24.3	25.7	.4744	-1.4 (-5.2 – 2.4)
Liver disease	12.3	11.6	.5879	0.8 (-2.1 – 3.7)
Peptic ulcer disease excluding bleeding	2.1	1.7	.5575	0.3 (-0.9 – 1.6)
AIDS/HIV	0.2	0.1	.5181[a]	0.1 (-0.3 – 0.4)
Lymphoma	2.5	1.5	.0953	0.9 (-0.4 – 2.3)
Metastatic cancer	4.9	4.6	.7323	0.3 (-1.6 – 2.2)
Solid tumor without metastasis	19.7	17.7	.2357	2.0 (-1.5 – 5.5)
Rheumatoid arthritis/collagen vascular diseases	11.2	9.9	.3227	1.3 (-1.4 – 4.1)
Coagulopathy	5.9	6.0	.9098	-0.1 (-2.2 – 1.9)
Obesity	25.8	23.7	.2631	2.1 (-1.7 – 6.0)
Weight loss	4.7	4.1	.4600	0.7 (-1.2 – 2.5)
Fluid and electrolyte disorders	7.0	8.1	.3870	-1.1 (-3.3 – 1.2)
Blood loss anemia	0.8	0.8	.9999[a]	0.0 (-0.8 – 0.7)
Deficiency anemia	7.4	9.0	.2006	-1.6 (-3.9 – 0.7)
Alcohol abuse	6.3	5.9	.7594	0.3 (-1.8 – 2.4)
Drug abuse	1.5	1.5	.9422	0.0 (-1.0 – 1.1)
Psychoses	2.1	3.0	.2232	-0.9 (-2.2 – 0.3)
Depression	33.6	32.1	.4697	1.5 (-2.6 – 5.7)

Abbreviations: CI, confidence interval; SD, standard deviation.
Notes: Boldface indicates significant differences (p<.05; confidence interval not including 0). [a] P-value calculated by using Fisher's exact test.

Table S2 Combined CONSORT 2010 and CONSORT-ROUTINE Checklist

Section/Topic	Item No.	CONSORT Extension for Trials Conducted using Cohorts or Routinely Collected Data Item	Reported on page #
Title and abstract			
	1a	Identification as a randomised trial in the title	1
	1b	Structured summary of trial design, methods, results, and conclusions (for specific guidance see CONSORT for abstracts). Specify that a cohort or routinely collected data were used to conduct the trial and, if applicable, provide the name of the cohort or routinely collected database(s)	1-2
Introduction			
Background and objectives	2a	Scientific background and explanation of rationale	2
	2b	Specific objectives or hypotheses	2
Methods			
Trial design	3a	Description of trial design (such as parallel, factorial) including allocation ratio, that a cohort or routinely collected database(s) was used to conduct the trial (such as electronic health record, registry) and how the data were used within the trial (such as identification of eligible trial participants, trial outcomes)	2-4
	3b	Important changes to methods after trial commencement (such as eligibility criteria), with reasons	N.A.
Cohort or routinely collected database	ROUTINE-1	Name, if applicable, and description of the cohort or routinely collected database(s) used to conduct the trial, including information on the setting (such as primary care), locations, and dates, (such as periods of recruitment, follow-up, and data collection)	2-4
	ROUTINE-2	Eligibility criteria for participants in the cohort or routinely collected database(s)	2
	ROUTINE-3	State whether the study included person-level, institutional-level, or other data linkage across two or more databases and, if so, linkage techniques and methods used to evaluate completeness and accuracy of linkage	3
Trial participants	4a	Eligibility criteria for trial participants, including information on how to access the list of codes and algorithms used to identify eligible participants, information on accuracy and completeness of data used to ascertain eligibility, and methods used to validate accuracy and completeness (e.g., monitoring, adjudication), if applicable	2

	4b	Settings and locations where the data were collected	2-4
	ROUTINE-4	Describe whether and how consent was obtained	2-3, 11
Interventions	5	The interventions for each group with sufficient details to allow replication, including how and when they were actually administered	2-3
Outcomes	6a	Completely defined pre-specified primary and secondary outcome measures, including how and when they were ascertained and the cohort or routinely collected database(s) used to ascertain each outcome	2-3
	ROUTINE-5	Information on how to access the list of codes and algorithms used to define or derive the outcomes from the cohort or routinely collected database(s) used to conduct the trial, information on accuracy and completeness of outcome variables, and methods used to validate accuracy and completeness (e.g., monitoring, adjudication), if applicable	N.A.
	6b	Any changes to trial outcomes after the trial commenced, with reasons	N.A
Sample size	7a	How sample size was determined	Czwikla et al. 2021
	7b	When applicable, explanation of any interim analyses and stopping guidelines	N.A.
Randomisation:			
Sequence generation	8a	Method used to generate the random allocation sequence	Czwikla et al. 2021
	8b	Type of randomisation; details of any restriction (such as blocking and block size)	Czwikla et al. 2021
Allocation concealment mechanism	9	Mechanism used to implement the random allocation sequence (such as embedding an automated randomiser within the cohort or routinely collected database(s)), describing any steps taken to conceal the sequence until interventions were assigned	Czwikla et al. 2021
Implementation	10	Who generated the random allocation sequence, who enrolled participants, and who assigned participants to interventions	2-3,<br>Czwikla et al. 2021
Blinding	11a	If done, who was blinded after assignment to interventions (for example, participants, care providers, those assessing outcomes) and how	2-3
	11b	If relevant, description of the similarity of interventions	N.A.
Statistical methods	12a	Statistical methods used to compare groups for primary and secondary outcomes	2-3,<br>Czwikla et al. 2021

	12b	Methods for additional analyses, such as subgroup analyses and adjusted analyses	4-5
Results			
Participant flow (a diagram is strongly recommended)	13a	For each group, the number of participants in the cohort or routinely collected database(s) used to conduct the trial and the numbers screened for eligibility, randomly assigned, offered and accepted interventions (e.g., cohort multiple RCTs), received intended treatment, and analysed for the primary outcome	2-3, Czwikla et al. 2021
	13b	For each group, losses and exclusions after randomisation, together with reasons	Czwikla et al. 2021
Recruitment	14a	Dates defining the periods of recruitment and follow-up	2-3
	14b	Why the trial ended or was stopped	N.A.
Baseline data	15	A table showing baseline demographic and clinical characteristics for each group	Table 1
Numbers analysed	16	For each group, number of participants (denominator) included in each analysis and whether the analysis was by original assigned groups	3, 5-7
Outcomes and estimation	17a	For each primary and secondary outcome, results for each group, and the estimated effect size and its precision (such as 95% confidence interval)	3, Czwikla et al. 2021
	17b	For binary outcomes, presentation of both absolute and relative effect sizes is recommended	N.A.
Ancillary analyses	18	Results of any other analyses performed, including subgroup analyses and adjusted analyses, distinguishing pre-specified from exploratory	5-7
Harms	19	All important harms or unintended effects in each group (for specific guidance see CONSORT for harms)	N.A.
Discussion			
Limitations	20	Trial limitations, addressing sources of potential bias, imprecision, and, if relevant, multiplicity of analyses	10-11
Generalisability	21	Generalisability (external validity, applicability) of the trial findings	7-11
Interpretation	22	Interpretation consistent with results, balancing benefits and harms, and considering other relevant evidence, including the implications of using data that were not collected to answer the trial research questions	7-11

Other information			
Registration	23	Registration number and name of trial registry	2
Protocol	24	Where the full trial protocol can be accessed, if available	N.A.
Funding	25	Sources of funding and other support for both the trial and the cohort or routinely collected database(s), role of funders	11

Please cite this as: Kwakkenbos L, Imran M, McCall SJ, et al. CONSORT extension for the reporting of randomised controlled trials conducted using cohorts and routinely collected data (CONSORT-ROUTINE): checklist with explanation and elaboration. *BMJ* 2021;373:n857.

Reference

Czwikla J, Herzberg A, Kapp S, Kloep S, Rothgang H, Nitschke I, Haffner C, Hoffmann F. Effectiveness of a Dental Intervention to Improve Oral Health among Home Care Recipients: A Randomized Controlled Trial. Int J Environ Res Public Health. 2021;18(17):9339.
